# Are We Missing Something About the Maximum Dosing of Botulinum Toxin Type A1 in Adult and Pediatric Patients with Spasticity?

**DOI:** 10.3390/toxins16120513

**Published:** 2024-11-27

**Authors:** Alessandro Picelli, Rita Di Censo, Stefano Tamburin, Nicola Smania, Mirko Filippetti

**Affiliations:** 1 Department of Neurosciences, Biomedicine and Movement Sciences, University of Verona, 37134 Verona, Italy; rita.dicenso@univr.it (R.D.C.); stefano.tamburin@univr.it (S.T.); nicola.smania@univr.it (N.S.); mirko.filippetti@univr.it (M.F.); 2 Department of Neurosciences, University Hospital of Verona, 37126 Verona, Italy; 3 Canadian Advances in Neuro-Orthopedics for Spasticity Consortium (CANOSC), Kingston, ON K7K 1Z6, Canada

**Keywords:** botulinum toxins, muscle spasticity, therapeutics

## Abstract

Botulinum toxin type A1 is a first-line treatment for adult and pediatric spasticity. However, when considering the quantity of 150 kDa neurotoxin protein in relation to patient weight and the maximum recommended dose for treating adult and pediatric patients with spasticity, several concerns arise. First, the therapeutic margin (the ratio of the actual maximum quantity of toxin recommended for treating adult spasticity to its median lethal dose) appears to be relevant. Second, there is no consistency between adult and pediatric dosing of botulinum toxin type A1 for spasticity. The third point concerns the suitability of the recommended doses for treating spasticity in pediatric patients. Based on the average body weight of American children and adolescents, the maximum weight-based doses for abobotulinumtoxinA and onabotulinumtoxinA could be administered to children as young as 9 years old. Additionally, the maximum weight-based dose for incobotulinumtoxinA could be administered to children as young as 6 years old. The final point concerns managing the maximum dose of BoNT/A1 in pediatric patients with spasticity who weigh more than 25 kg for incobotulinumtoxinA, or more than 34 kg for abobotulinumtoxinA and onabotulinumtoxinA. No labeled recommendations are given on the weight cut-off for transitioning to adult dosing in pediatric patients.

## 1. Introduction

Clostridium is a genus of sporulating and anaerobic gram-positive bacteria [1,2]. Their spores can withstand physical and chemical stress for extended periods until favorable environmental conditions, such as humidity, nutrients, and the absence of oxygen, enable germination [1]. Clostridium botulinum is one of the few neurotoxigenic species within the genus [1,2]. To date, eight serotypes of botulinum neurotoxins (BoNTs) have been identified, labeled A, B, C, D, E, F, G, and X [2]. Recent advancements in molecular genetic analysis and sequencing techniques have led to the identification of several BoNT subtypes, designated by a number following the serotype letter [3].

The molecular architecture of all BoNT serotypes is similar, consisting of a single-chain polypeptide of 150 kDa [2,3]. The toxin is produced by clostridium botulinum as an inactive protein that requires proteolytic cleavage to become active [3]. The active BoNT molecule consists of a light (L) chain (50 kDa) and a heavy (H) chain (100 kDa) connected by a disulfide bond [2,3,4].

BoNTs exert their effects through a four-step mechanism: binding, internalization, membrane translocation, and enzymatic target modification [4]. The C-terminal of the H chain is primarily responsible for strong neurospecific binding with the unmyelinated areas of peripheral cholinergic nerve endings, while the N-terminal of the H chain facilitates the translocation of the L chain across the endocytic vesicle membrane into the cytosol [3,4]. The intracellular catalytic activity of the toxin is due to the L chain [1,2,3,4]. SNARE proteins, specifically VAMP/synaptobrevin, SNAP-25, and syntaxin, mediate vesicle docking and membrane fusion to release neurotransmitters into the synaptic cleft [2,3,4]. These proteins are specific substrates for the L chain metalloprotease catalytic activity, which selectively cleaves SNARE proteins at different peptide bonds, thereby inhibiting neurotransmitter release into the synaptic cleft [2,3].

## 2. Botulinum Toxin Type A1 in Spasticity Clinics

Research into the potential clinical use of BoNTs began in the 1970s, with pioneering studies conducted by Alan Brown Scott and his colleagues [5]. In 1989, the Food and Drug Administration (FDA) approved the clinical use of BoNT/A1 for the first time as a treatment for blepharospasm and strabismus in adults [5,6]. Today, BoNT/A1 and BoNT/B1 are widely used in clinical practice for various therapeutic indications [2,6].

Only BoNT/A1 has been approved for the treatment of spasticity in both adult and pediatric patients [5,6,7,8]. The endocytosis of BoNT/A is facilitated by a specific receptor called SV protein, which is expressed at motor nerve terminals in three isoforms (A, B, and C) [3,4]. SV2C binds the toxin more efficiently than SV2A and SV2B [2,3]. BoNT/A1 selectively targets SNAP-25, generating cleavage fragments (SNAP-25_1–197_) that inhibit acetylcholine exocytosis at the neuromuscular junction, leading to a neuromuscular blocking effect [2,3,4]. The duration of BoNT/A1 action is approximately 3–4 months in humans, necessitating repeated toxin injections in cycles [2,3,7].

Currently, three major branded products containing BoNT/A1 are available worldwide for the treatment of spasticity: abobotulinumtoxinA, marketed as Dysport by Ipsen (Boulogne-Billancourt, France); onabotulinumtoxinA, marketed as Botox by AbbVie Inc. (North Chicago, IL, USA); and incobotulinumtoxinA, marketed as Xeomin by Merz Pharmaceutical GmbH (Frankfurt, Germany) [9]. As shown in Table 1, these products have different characteristics and should not be considered interchangeable [9,10].

Despite its well-documented effectiveness in reducing spasticity, the response to BoNT/A1 may be unsatisfactory in some cases when treatment goals are not met [11,12]. Potential reasons for a poor outcome in BoNT/A1 therapy for spasticity can be related to the patient (e.g., unrealistic expectations, underlying medical conditions, interactions with concurrent medications, immunoresistance, secondary muscle changes), the injector (e.g., incorrect diagnosis, improper muscle selection, poor injection technique), or the drug itself (e.g., incorrect dose, improper preparation, inactive medication) [11].

## 3. Botulinum Toxin Type A1 for Treating Spasticity

Spasticity is a positive symptom of upper motor neuron syndrome (UMNS). Depending on the various etiologies of UMNS (such as stroke, multiple sclerosis, brain injury, spinal cord injury, or cerebral palsy), spasticity may present with different features, necessitating specific therapeutic approaches [13]. BoNT/A1 is a first-line treatment for focal spasticity [8,12]. However, the labeling information for major BoNT/A1 products is not consistent worldwide, as it varies between countries depending on regulatory approvals [14,15]. For example, according to current labeling information, abobotulinumtoxinA and onabotulinumtoxinA are approved in the USA for the treatment of upper- and lower-limb spasticity in both adults and children [14]. By contrast, the Agenzia Italiana del Farmaco (AIFA) has approved these products in Italy for the treatment of upper- and lower-limb spasticity in adults (with onabotulinumtoxinA specifically approved only for post-stroke spasticity) and for the treatment of lower-limb spasticity due to cerebral palsy in children [15]. IncobotulinumtoxinA is approved in the USA for the treatment of upper-limb spasticity in adults and children (excluding spasticity caused by cerebral palsy) [14]. In Italy, however, it is approved only for the treatment of upper-limb spasticity in adults, with no approval for pediatric spasticity [15]. Additionally, current FDA and AIFA labeling information for the treatment of spasticity includes some differences in recommendations regarding the doses and target muscles for injections with abobotulinumtoxinA (see Table 2), incobotulinumtoxinA (see Table 3), and onabotulinumtoxinA (see Table 4) [14,15].

While the dose per muscle is influenced by factors such as muscle tone severity, muscle characteristics, and architecture, the primary factor in determining the amount of BoNT/A1 to administer per treatment session is the maximum recommended dose, which defines the total number of patterns and muscles that can be injected. This is a major concern, as a more tailored treatment approach may improve outcomes and better support neurorehabilitation programs.

Incorrect dosing, whether overdosing or underdosing, is a key drug-related factor contributing to poor outcomes in BoNT/A1 therapy [11]. The current literature suggests that many patients with spasticity feel that BoNT/A1 dosing limits hinder both outcome achievement and treatment satisfaction. Similarly, physicians are often required to prioritize treating the patterns that most significantly impact overall treatment goals [16]. Therefore, addressing the consistency and discrepancies in approved BoNT/A1 dosages is essential.

## 4. Discussion

A major concern in managing spasticity with BoNT/A1 is the potential for local and systemic adverse events [17,18]. In clinical practice, the safety of BoNT/A1 treatment is closely tied to the administered dose [10,18]. Dose-related issues across different products can have serious consequences for patients: doses that are too low may not achieve optimal efficacy, while excessive doses can increase the risk of adverse events [10].

Current labeling information about the maximum recommended total dose (units) per treatment session of abobotulinumtoxinA, incobotulinumtoxinA, and onabotulinumtoxinA is reported in Table 5.

When considering the amount of BoNT/A1 (150 kDa neurotoxin protein) per vial, the mean quantity of toxin is 2.69 ng/500 units of abobotulinumtoxinA, 0.40 ng/100 units of incobotulinumtoxinA, and 0.90 ng/100 units of onabotulinumtoxinA [19]. Table 6 compares the maximum recommended total dose of the three major branded products converted in ng.

BoNTs are among the most potent toxins known to science, with toxicity measured in ng/kg. The toxicity of BoNT/A1 in humans is estimated to be 1 ng/kg of body weight when administered intramuscularly or intravenously [20].

Based on this, one would expect the therapeutic dosage of BoNT/A1 to also be calculated according to body weight. However, for the treatment of spasticity with BoNT/A1, the maximum recommended total dose is calculated based on body weight (units/kg) only for pediatric patients aged 2 to 17 years (see Table 5). Thus, according to the FDA and AIFA labeling information, the maximum weight-based total doses for abobotulinumtoxinA (1000 units/30 units per kg) and onabotulinumtoxinA (360 units/10 units per kg or 200 units/6 units per kg) can be administered to children weighing 34 kg [14,15]. Additionally, the maximum weight-based total dose for incobotulinumtoxinA (400 units/16 units per kg) can be administered to children weighing 25 kg [14]. By contrast, for adults with spasticity, there is no specific recommendation for BoNT/A1 total dosing adjusted by body weight. However, considering the average body weight of American adults aged over 20 years [21], the maximum total dose of BoNT/A1 recommended by FDA labeling corresponds to 16.6 units/kg (1500 units/90.6 kg) in males and 19.4 units/kg (1500 units/77.5 kg) in females for abobotulinumtoxinA, and 4.4 units/kg (400 units/90.6 kg) in males and 5.2 units/kg (400 units/77.5 kg) in females for both incobotulinumtoxinA and onabotulinumtoxinA [14,21]. For comparison, we report these conversions to ng/kg of the maximum BoNT/A1 total doses recommended by the FDA for treating spasticity in adults and pediatric patients in Table 7.

Overall, when considering the quantity of BoNT/A1 (150 kDa neurotoxin protein) in relation to patient weight and the maximum recommended total dose for treating adult and pediatric patients with spasticity, several concerns arise.

First, the therapeutic margin (i.e., the difference between the therapeutic dose and the toxic dose) appears to be relevant. Specifically, when adjusting the maximum recommended total dose of toxin based on the average body weight of the American adult population, the ratio of the actual maximum quantity of BoNT/A1 recommended by the FDA for treating adult spasticity to its median lethal dose is approximately 9–10% for abobotulinumtoxinA, 2–3% for incobotulinumtoxinA, and 4–5% for onabotulinumtoxinA. To the best of our knowledge, doses up to 2000 units of abobotulinumtoxinA, 1200 units of incobotulinumtoxinA, and 800 units of onabotulinumtoxinA have been reported in adult patients with spasticity [22,23,24]. Based on the parameters previously considered, these total doses correspond to 0.12–0.14 ng/kg (12–14% of the median lethal dose) for abobotulinumtoxinA, 0.05–0.06 ng/kg (5–6% of the median lethal dose) for incobotulinumtoxinA, and 0.08–0.09 ng/kg (8–9% of the median lethal dose) for onabotulinumtoxinA. The current literature reports no significant increase in the risk for adverse events with these (high) doses of BoNT/A1, further suggesting that the current approved doses should be reconsidered [22,23,24,25].

Second, although adult doses appear consistent between males and females, with only a very small difference of 0.01 ng/kg when adjusted for the average body weight of the American population, there is no consistency between the adult and pediatric maximum total dosing for spasticity [21,26]. Specifically, the maximum pediatric dose recommended by the FDA for treating spasticity is approximately 60–78% higher than that for adults for abobotulinumtoxinA, 200–300% higher for incobotulinumtoxinA, and 180–225% higher for onabotulinumtoxinA.

This leads to the third point, which concerns the suitability of the recommended doses for treating spasticity in pediatric patients aged 2 to 17 years. Based on the average body weight of American children and adolescents [26], the maximum weight-based total doses for abobotulinumtoxinA (1000 units/30 units per kg) and onabotulinumtoxinA (340 units/10 units per kg) could be administered to children as young as 9 years old. Additionally, the maximum weight-based total dose for incobotulinumtoxinA (400 units/16 units per kg) could be administered to children as young as 6 years old [14,26]. This suggests that, according to the current FDA labeling for pediatric spasticity [14], a 6 year-old girl weighing 25 kg would receive the same maximum total dose of incobotulinumtoxinA as an adult female weighing 77 kg. Similarly, a 9 year-old boy weighing 34 kg would receive the same maximum total dose of abobotulinumtoxinA and onabotulinumtoxinA as an adult male weighing 90 kg [14,21,26].

The final point concerns managing the maximum total dose of BoNT/A1 in pediatric patients with spasticity who weigh more than 25 kg for incobotulinumtoxinA, or more than 34 kg for abobotulinumtoxinA and onabotulinumtoxinA. Unfortunately, there are no labeled recommendations on the weight cut-off for transitioning to adult dosing in pediatric patients. Previous expert opinion suggested using adult dosing of onabotulinumtoxinA for children weighing more than 60 kg, which corresponds to the average weight of 13 year-olds in the USA [26,27]. However, the recent consensus no longer supports this approach, instead endorsing the in-label maximum total doses of BoNT/A1, with the exception of allowing up to 600 units of onabotulinumtoxinA in specific pediatric patients [28].

## 5. Conclusions

Treatment of spasticity with BoNT/A1 has been shown to be effective and safe in both adult and pediatric patients [8,9,10,11,12]. However, the current maximum recommended dose for spasticity appears inconsistent, as pediatric patients are advised to receive a higher maximum dose than adults, and adult females are prescribed higher maximum doses of BoNT/A1 than adult males when adjusted for body weight [14,21,26]. Given that BoNT toxicity is measured in ng/kg and the incidence of systemic adverse events following BoNT/A1 treatment is similar across adult males, females, and pediatric patients with spasticity [29,30], there may be a need to revise the recommended maximum doses according to the actual quantity (ng) of the 150 kDa neurotoxin protein injected and patient characteristics. From this perspective, implementing alternative therapeutic strategies, such as an individualized approach optimized on the base of body weight and other contextual factors (such as muscle mass, spasticity etiology, disease duration, previous treatments, comorbidities) [31], could help improve treatment consistency, particularly between children and adults. Addressing these dosage discrepancies in labeling information may significantly impact treatment options for patients with spasticity.

## Figures and Tables

**Table 1:** Main characteristics of branded products containing BoNT/A1.

	abobotulinumtoxinA	incobotulinumtoxinA	onabotulinumtoxinA
Brand name	Dysport	Xeomin	Botox
Units per vial	300; 500	50; 100; 200	100; 200
Complex size	~500 kDa	150 kDa	900 kDa
Preparation	Lyophilized	Lyophilized	Vacuum-dried
Storage	2–8 °C	<25 °C	2–8 °C
Constituents and excipients	Hemagglutinin, human albumin, lactose	Human albumin, saccharose	Hemagglutinin, human albumin, saccharose, NaCl

**Table 2:** Recommendations for the treatment of spasticity with abobotulinumtoxinA.

	USA (Food and Drug Administration)	Italy (Agenzia Italiana del Farmaco)
Upper-limb spasticity in adults	Biceps brachii *200–400 units* Brachialis *200–400 units* Brachioradialis *100–200 units* Pronator teres *100–200 units* Flexor carpi radialis *100–200 units* Flexor carpi ulnaris *100–200 units* Flexor digitorum superficialis *100–200 units* Flexor digitorum profundus *100–200 units*	Latissimus dorsi *150–300 units* Subscapolaris *150–300 units* Pectoralis major *150–300 units* Triceps brachii *150–300 units* Biceps brachii *200–400 units* Brachialis *200–400 units* Brachioradialis *100–200 units* Pronator teres *100–200 units* Flexor carpi radialis *100–200 units* Flexor carpi ulnaris *100–200 units* Flexor digitorum superficialis *100–200 units* Flexor digitorum profundus *100–200 units* Flexor pollicis longus *100–200 units* Adductor pollicis *25–50 units*
Lower-limb spasticity in adults	Gastrocnemius medialis *100–150 units* Gastrocnemius lateralis *100–150 units* Soleus *330–500 units* Tibialis posterior *200–300 units* Flexor digitorum longus *130–200 units* Flexor hallucis longus *70–200 units*	Gluteus maximum *100–400 units* Gracilis *100–200 units* Adductor magnus *100–300 units* Rectus femoris *100–400 units* Hamstrings *100–400 units* Gastrocnemius medialis *100–450 units* Gastrocnemius lateralis *100–450 units* Soleus *300–550 units* Tibialis posterior *100–250 units* Flexor digitorum longus *50–200 units* Flexor digitorum brevis *50–200 units* Flexor hallucis longus *50–200 units* Flexor hallucis brevis *50–100 units*
Upper-limb spasticity in children	Biceps brachii *3–6 units/kg* Brachialis *3–6 units/kg* Brachioradialis *1.5–3 units/kg* Pronator teres *1–2 units/kg* Pronator quadratus *0.5–1 units/kg* Flexor carpi radialis *2–4 units/kg* Flexor carpi ulnaris *1.5–3 units/kg* Flexor digitorum superficialis *1.5–3 units/kg* Flexor digitorum profundus *1–2 units/kg*	Not approved
Lower-limb spasticity in children	Gastrocnemius *6–9 units/kg* Soleus *4–6 units/kg*	Hip adductors *3–10 units/kg* Hamstrings *5–6 units/kg* Gastrocnemius *5–15 units/kg* Soleus *4–6 units/kg* Tibialis posterior *3–5 units/kg*

**Table 3:** Recommendations for the treatment of spasticity with incobotulinumtoxinA.

	USA (Food and Drug Administration)	Italy (Agenzia Italiana del Farmaco)
Upper-limb spasticity in adults	Biceps brachii *50–200 units* Brachialis *25–100 units* Brachioradialis *25–100 units* Pronator teres *25–75 units* Pronator quadratus *10–50 units* Flexor carpi radialis *25–100 units* Flexor carpi ulnaris *20–100 units* Flexor digitorum superficialis *25–100 units* Flexor digitorum profundus *25–100 units* Flexor pollicis longus *10–50 units* Adductor pollicis *5–30 units* Flexor pollicis brevis *5–30 units* Opponens pollicis *5–30 units*	Latissimus dorsi *25–150 units* Deltoid *20–150 units* Subscapolaris *15–100 units* Pectoralis major *20–200 units* Teres major *20–100 units* Biceps brachii *50–200 units* Brachialis *25–100 units* Brachioradialis *25–100 units* Pronator teres *25–75 units* Pronator quadratus *10–50 units* Flexor carpi radialis *25–100 units* Flexor carpi ulnaris *25–100 units* Flexor digitorum superficialis *25–100 units* Flexor digitorum profundus *25–100 units* Flexor pollicis longus *10–50 units* Adductor pollicis *5–30 units* Flexor pollicis brevis *5–30 units* Opponens pollicis *5–30 units*
Upper-limb spasticity in children	Biceps brachii *2–3 units/kg* Brachialis *1–2 units/kg* Brachioradialis *1–2 units/kg* Pronator teres *1–2 units/kg* Pronator quadratus *0.5 units/kg* Flexor carpi radialis *1 units/kg* Flexor carpi ulnaris *1 units/kg* Flexor digitorum superficialis *1 units/kg* Flexor digitorum profundus *1 units/kg* Flexor pollicis longus *1 units/kg* Adductor pollicis *0.5 units/kg* Flexor pollicis brevis *0.5 units/kg* Opponens pollicis *0.5 units/kg*	Not approved

**Table 4:** Recommendations for the treatment of spasticity with onabotulinumtoxinA.

	USA (Food and Drug Administration)	Italy (Agenzia Italiana del Farmaco)
Upper-limb spasticity in adults	Biceps brachii *60–200 units* Brachialis *30–50 units* Brachioradialis *45–75 units* Pronator teres *15–25 units* Pronator quadratus *10–50 units* Flexor carpi radialis *12.5–50 units* Flexor carpi ulnaris *12.5–50 units* Flexor digitorum superficialis *30–50 units* Flexor digitorum profundus *30–50 units* Flexor pollicis longus *20 units* Adductor pollicis *20 units* Flexor pollicis brevis *5–25 units* Opponens pollicis *5–25 units* Lumbricals *5–10 units* Interossei *5–10 units*	Pronator teres *10–50 units* Flexor carpi radialis *15–60 units* Flexor carpi ulnaris *10–50 units* Flexor digitorum superficialis *10–50 units* Flexor digitorum profundus *10–50 units* Flexor pollicis longus *20 units* Adductor pollicis *20 units* Flexor pollicis brevis *5–25 units* Opponens pollicis *5–25 units* Lumbricals *5–10 units* Interossei *5–10 units*
Lower-limb spasticity in adults	Gastrocnemius medialis *75 units* Gastrocnemius lateralis *75 units* Soleus *75 units* Tibialis posterior *75 units* Flexor digitorum longus *50 units* Flexor hallucis longus *50 units*	Gastrocnemius medialis *75 units* Gastrocnemius lateralis *75 units* Soleus *75 units* Tibialis posterior *75 units*
Upper-limb spasticity in children	Biceps brachii *1.5–3 units/kg* Brachialis *1–2 units/kg* Brachioradialis *0.5–1 units/kg* Flexor carpi radialis *1–2 units/kg* Flexor carpi ulnaris *1–2 units/kg* Flexor digitorum superficialis *0.5–1 units/kg* Flexor digitorum profundus *0.5–1 units/kg*	Not approved
Lower-limb spasticity in children	Gastrocnemius medialis *1–2 units/kg* Gastrocnemius lateralis *1–2 units/kg* Soleus *1–2 units/kg* Tibialis posterior *1–2 units/kg*	Gastrocnemius *4–6 units/kg*

**Table 5:** Maximum recommended doses (units) of BoNT/A1 for treating spasticity.

	abobotulinumtoxinA	incobotulinumtoxinA	onabotulinumtoxinA
Adults	USA *1500 units* Italy *1500 units*	USA *400 units* Italy *500 units*	USA *400 units* Italy *300 units*
Children	USA *1000 units (30 units/kg)* Italy *1000 units (30 units/kg)*	USA *400 units (16 units/kg)* Italy *not approved*	USA *340 units (10 units/kg)* Italy *200 units (6 units/kg)*

**Table 6:** Maximum recommended doses (ng) of BoNT/A1 for treating spasticity.

	abobotulinumtoxinA	incobotulinumtoxinA	onabotulinumtoxinA
Adults	USA *8.07 ng* Italy *8.07 ng*	USA *1.6 ng* Italy *2.0 ng*	USA *3.6 ng* Italy *2.7 ng*
Children	USA *5.38 ng* Italy *5.38 ng*	USA *1.6 ng* Italy *not approved*	USA *3.06 ng* Italy *1.8 ng*

**Table 7:** Maximum doses (ng/kg) recommended by FDA for treating spasticity.

	abobotulinumtoxinA	incobotulinumtoxinA	onabotulinumtoxinA
Adult males	*0.09 ng/kg*	*0.02 ng/kg*	*0.04 ng/kg*
Adult females	*0.10 ng/kg*	*0.03 ng/kg*	*0.05 ng/kg*
Children	*0.16 ng/kg*	*0.06 ng/kg*	*0.09 ng/kg*

## Data Availability

No new data were created or analyzed in this study. Data sharing is not applicable to this article.
